# Brain and lung arteriovenous malformation rescreening practices for children and adults with hereditary hemorrhagic telangiectasia

**DOI:** 10.1186/s13023-024-03402-8

**Published:** 2024-11-09

**Authors:** Lauren A. Beslow, Helen Kim, Steven W. Hetts, Felix Ratjen, Marianne S. Clancy, James R. Gossage, Marie E. Faughnan

**Affiliations:** 1 Neurology and Pediatrics, Children’s Hospital of Philadelphia and Perelman School of Medicine at the University of Pennsylvania, 3401 Civic Center Boulevard, Philadelphia, PA 19104 USA; 2 University of California San Francisco, San Francisco, CA USA; 3 Department of Radiology & Biomedical Imaging and Neurological Surgery, Division of NeuroEndovascular Surgery, University of California San Francisco, San Francisco, CA USA; 4 Paediatrics and Paediatric Respiratory Medicine, The Hospital for Sick Children and University of Toronto, Toronto, ON Canada; 5 Cure HHT Foundation, Monkton, MD USA; 6 Division of Pulmonary, Critical Care, and Sleep Medicine, Augusta University, Augusta, GA USA; 7 Toronto HHT Centre, Division of Respirology, St. Michael’s Hospital, Li Ka Shing Knowledge Institute and University of Toronto, Toronto, ON Canada

**Keywords:** Hereditary hemorrhagic telangiectasia, Arteriovenous malformation, Rescreen, Reimage, Magnetic resonance imaging, Echocardiography

## Abstract

**Background:**

Patients with hereditary hemorrhagic telangiectasia (HHT) are at risk for organ vascular malformations including arteriovenous malformations (AVMs) in the brain and lungs. North American HHT Centers of Excellence (CoEs) routinely screen for brain and lung AVMs, with the primary goal of detecting AVMs which can be treated before complications arise. Current international HHT guidelines provide recommendations for initial screening for brain and lung AVMs among children and adults with the disease, but rescreening recommendations are not comprehensively addressed and have not been reported. We determined current rescreening practices for brain and lung AVMs for children and adults with HHT among North American HHT CoEs.

**Methods:**

We surveyed North American HHT CoEs regarding rescreening practices for new brain and lung AVMs in children and adults with initial negative screening.

**Results:**

All thirty CoEs responded; 28 regarding pediatric (93.3%) and 30 (100%) regarding adult HHT care. The median duration of practice experience in HHT was 11.5 (range 3–30) years for providers of pediatric HHT care and 11.5 (range 3–35) years for providers of adult HHT care. The median number of patients followed at each CoE was 60 for children (range 8–500) and 375 for adults (range 30–1500). 25/28 CoEs (89.3%) reported rescreening children for brain AVMs, most commonly with enhanced MRI (21/25, 84%). 25 CoEs rescreen children for lung AVMs, most commonly every 5 years (15/25). Only 4/30 CoEs (13.3%) rescreen adults for brain AVMs. 26/30 CoEs (86.7%) reported rescreening adults for lung AVMs, most commonly every 5 years (18/26, 69.2%).

**Conclusions:**

Most HHT CoEs routinely rescreen children for brain and lung AVMs and adults for lung AVMs when initial screening is negative, but adults are infrequently rescreened for brain AVMs. Long-term data regarding risk for new brain and lung AVMs are required to establish practice guidelines for rescreening.

**Supplementary Information:**

The online version contains supplementary material available at 10.1186/s13023-024-03402-8.

## Background

Hereditary hemorrhagic telangiectasia (HHT) is an autosomal dominant vascular disease affecting approximately 1 in 5,000–10,000 people worldwide [1, 2]. Patients with HHT are at risk for organ vascular malformations including in the brain and lungs. Brain AVMs place patients at risk for intracerebral hemorrhage, and pulmonary AVMs place patients at risk for embolic ischemic stroke and brain abscess. Screening for organ AVMs in people with HHT is important to reduce risk of major complications. One study demonstrated that survival among HHT patients who were screened for AVMs and subsequently treated at HHT Centers of Excellence (CoEs), if treatment was required, had comparable survival to patients without HHT [3]. The Second International Guidelines for the Diagnosis and Management of HHT provide clear guidance with excellent consensus for initial screening for brain AVMs in children with the disease (using contrast enhanced brain magnetic resonance imaging [MRI]) and for initial screening for lung AVMs in children and adults with the disease (using contrast echocardiogram for children and adults with the option of a combination of chest X-ray and oxygen saturation in children) [4, 5]. However, international consensus was not reached for routine brain AVM screening in adults [4]. Kilian et al. surveyed 28 North American HHT CoEs about standard practice for initial AVM screening of adults and children with HHT and found that despite lack of international consensus about brain AVM screening for adults, 100% of North American CoEs screened adults for brain AVMs in addition to screening for lung AVMs [6]. For children, 89% of CoEs performed initial screening for brain AVMs, and 82% performed initial screening for lung AVMs.

The focus of the HHT guidelines is initial screening and management, and these guidelines do not provide guidance for rescreening for brain and lung AVMs in HHT, with the exception of a recommendation to rescreen asymptomatic children for lung AVMs every 5 years [4]. While there are reports of children developing de novo (new) brain vascular malformations [7–10] and also of small lung AVMs enlarging over time [11], there is a paucity of long-term clinical and serial imaging data that identify the risk of de novo brain and lung AVM formation among patients with HHT, limiting rescreening practice recommendations. We aimed to report rescreening practices for brain and lung AVMs for children and adults at 30 North American HHT CoEs and to understand related decision making.

## Methods

We designed a survey for HHT CoEs (Supplemental Fig. 1) regarding rescreening practices for brain AVMs and lung AVMs for children and adults and distributed it via SurveyMonkey. Questions addressed the following topic areas: (1) physician and center HHT experience, (2) rescreening practices including time intervals and duration of screening, (3) rescreening modality for brain and lung AVMs, and (4) rationale for rescreening or not rescreening. Additional questions were included for rescreening practices for patients with a known brain or lung AVM that no longer required surveillance (e.g., brain AVM has been fully resected with confirmed lack of residual AVM). Most questions allowed for additional text comments. The survey was reviewed with additional modifications made by several physician authors from different specialties (LAB, SWH, FR, MEF) and a patient advocacy representative (MSC). The final survey was distributed to HHT CoE directors by Cure HHT (cureHHT.org) and was open from August 9, 2022 to January 17, 2023 to ensure that all centers had ample time to respond. Center directors who primarily care for adults were able to request that the survey be sent to a pediatric counterpart at the CoE. In this case, the center director had the option to answer the questions or leave them unfilled. When a respondent who primarily cares for adult patients but evaluates some pediatric patients responded to pediatric questions but another primarily pediatric expert also responded from that site, we used the responses from the pediatric expert for that site as these were considered most reflective of the CoE’s practices. Research ethics board approval was not required because no patients were involved, responses were voluntary, and individual and site-specific responses are not reported.

## Results

All thirty North American HHT CoEs responded to the survey; 28 about pediatric care (93%) and 30 about adult HHT care (100%). A pediatric expert responded to the pediatric questions at 17 CoEs; pediatric questions were answered by an adult expert who manages both adults and children with HHT at 11 CoEs. Questions about adult care were answered by an adult expert at 29 CoEs and by a pediatric-trained expert at one CoE at which the center director manages adults and children.

Among the 28 pediatric HHT care experts, the median duration of HHT practice experience was 11.5 years (interquartile range [IQR] 7.5–17.5 years, range 3–30 years). The median number of children followed was 60 per center (IQR 30–143 children, range 8–500 children). Among the 30 adult HHT care experts, the median duration of HHT practice experience was 11.5 years (IQR 8–18 years, range 3–35 years). The median number of adults followed was 375 per center (IQR 140–500 adults, range 30–1500 adults). The main survey findings are presented in Table 1.

**Table 1:** Summary of rescreening survey findings

Pediatric care responses (28)*	Adult care responses (30)
25/28 (89.3%) rescreen for brain AVMs in patients with prior negative imaging • 21/25 with contrast enhanced MRI • 16/25 once or twice; 9/25 every 5 or 10 years (most until age 18–25 years) • Most common reason for rescreening: to detect growth of brain AVMs too small to detect on initial imaging	4/30 (13.3%) rescreen for brain AVMs in patients with prior negative imaging • 3/4 with contrast enhanced MRI • Two reimage once, one at 5-year intervals, one at 10-year intervals • Most common reason for not rescreening: minimal risk of development of new brain AVMs in adults
25/27 (92.6%) rescreen for lung AVMs in patients with prior negative screening • 18/25 with contrast echocardiography • 18/25 every 5 years • Most common reason for rescreening: literature description of patients developing new lung AVMs	26/30 (86.7%) rescreen for lung AVMs in patients with prior negative screening • 26/26 with contrast echocardiography • 18/25 every 5 years • Most common reason for rescreening: to detect growth of lung AVMs too small to be identified on initial screening

^*^Twenty-eight centers answered questions about pediatric brain AVMs, but 27 answered questions about pediatric lung AVMs

### Brain AVM rescreening practices

#### Pediatric brain AVMs

Rescreening practices for children with HHT in whom initial imaging was negative for brain AVM and in whom no brain AVM symptoms exist are reported in Table 2. Of 28 pediatric centers, 25 (89.3%) rescreen children routinely, most often with contrast enhanced MRI (21/25, 84%). One of three centers that does not routinely rescreen all children with HHT once previous imaging was negative indicated that children are rescreened if the initial MRI was performed without contrast. Eight centers always include an MRA head in their rescreening protocol, while seven centers sometimes include an MRA head. Sixteen centers rescreen one or two times, most often in adolescence or prior to transfer to an adult center. Six centers rescreen every 5 years, and three rescreen every 10 years. For the nine centers that rescreen at five- or ten-year intervals, six rescreen until age 18–25 years, though one center rescreens throughout the lifespan.

**Table 2:** Pediatric brain AVM rescreening practices for children without symptoms of brain AVM and with initial brain imaging negative for brain AVM

Routinely Reimage (25) •Every 5 years (6) ◦Until age 18 (3) ◦Until age 25 (1) ◦If first image was performed at < 5 years every 5 years, if at between 5 and 10 years every 5–10 years, if at > 10 years every 10 years (1) ◦Considers age at first image, quality, need for gadolinium (1) •Every 10 years (3) ◦Lifetime (1) ◦Once as preteen, once as adult then stop if adult imaging negative (1) ◦At age 6 months, age 10 years, age 18–20 years (1) •Re-image once or twice (16) ◦If initial image performed at age < 2 years (1) ◦If initial imaging at < 5 years (1) ◦Post puberty and age 17–18 years or prior to transition to adult care (2) ◦If initial image performed prior to adolescence/puberty (3) ◦As late teen or young adult (1) ◦In mid adolescence/as teenagers (2) ◦At age 18 years to young adult (4) ▪No matter the age at initial image (3); also considers image at age 9–10 years in those with family history of brain AVM ◦Prior to transition to adult care (1) ◦As an adult (1) Rescreening Modality •MRI enhanced (21) •MRI unenhanced (3) •No response (1) •MRA always (8), sometimes (9), never (7), no response (1) obtained with MRI	Do Not Routinely Reimage (3) •Rescreen only if initial MRI performed without contrast (1) •All 3 who responded no to rescreening are adult-trained experts who routinely treat children with HHT

Rationale for pediatric rescreening practices for brain AVM evaluation are found in Table 3, and centers could choose multiple reasons for rescreening or not. The most common reason cited for rescreening for brain AVMs in children with prior negative screening was to detect growth of brain AVMs that were too small to detect on initial imaging (18/25, 72%). Fourteen centers cited literature that describes patients who developed new brain AVMs, nine cited personal experience with patients developing new brain AVMs, and six noted that a colleague had a patient with a new brain AVM. Eleven centers sought to reassure parents and patients. Two of three centers that did not rescreen cited reasons for not rescreening, and both cited a minimal risk of new brain AVM formation in children and minimal yield of repeat imaging. No center cited concerns about sedation, causing unnecessary worry to parents or children, cost considerations, or insurance approval as reasons for not rescreening.

**Table 3:** Rationale for rescreening and for not rescreening for brain AVMs in children with HHT with initial negative brain imaging and no symptoms of brain AVM

Rationale for rescreening (25 Respondents)	Rationale for not rescreening (2 Respondents)
Reassure parents/patients (11)	Cause patient/family unnecessary worry (0)
Literature describes patients who developed new brain AVM (14)	Minimal to no risk of new brain AVM formation in CHILDREN (2)
Personal experience with patient developing new brain AVM (9)	In my own experience/in my center's experience, the yield of rescreening in CHILDREN with previous negative screening for brain AVM is too low (2)
Colleague had experience with patient developing new brain AVM (6)	In my own experience/in my center’s experience, the evidence for treatment of asymptomatic brain AVMs in CHILDREN is not sufficient to warrant rescreening in CHILDREN with previous negative screening (0)
Detect brain AVM(s) that were too small to detect on initial imaging that have grown (18)	Cost considerations (0)
Other (7) •Prior to transitioning to adult care (1) •Part of a prospective study assessing whether new AVMs do develop in children, and/or at what age they develop, and/or how quickly they develop (1) •We do not have the data yet to ensure that no new AVMs will develop (1) •Current guidelines (1) •Neurosurgery input (1) •Improvements may occur in MRI technology over the roughly 20 years between the first and last MRI that I might obtain (1) •Technology is changing—some may have been there at/below level of detection previously (1)	Difficulty with insurance approval (0)
	Need for sedation (0)

Respondents were able to choose more than one rationale. One of three centers that do not routinely reimage for brain AVMs did not respond to this question

Rescreening practices for new brain AVMs in children with HHT in whom initial imaging demonstrated a brain AVM but for whom follow-up imaging for the previously diagnosed brain AVM is no longer required are presented in Supplemental Table 1. Twenty-three of 26 (88.5%) respondents routinely obtain repeat imaging under these circumstances, while three centers do not. One center rescreens every two years, six every five years, three every 10 years, and eight rescreen once or twice. Of the ten that rescreen periodically, seven rescreen until between age 18 and 25 years. Four centers defer to neurosurgery or neurointerventional specialists to determine frequency of rescreening, and one center’s rescreening protocol for children with treated brain AVMs varies depending on the child.

#### Adult brain AVMs

Rescreening practices for adults with HHT in whom initial imaging was negative for brain AVM and in whom no brain AVM symptoms exist are reported in Table 4. Only four of 30 centers (13.3%) routinely reimage adults for development of new brain AVMs. Of those four, two reimage once and two at intervals, every five years and every ten years throughout the lifespan, respectively. Of the 26 that do not rescreen, three commented that there are certain circumstances for which rescreening is considered, including MRI performed a long time ago or without contrast. Of the four centers that rescreen, three perform enhanced MRI, and the other performs MRI with arterial spin labelling. Two centers always include an MRA head, and one center sometimes includes an MRA head.

**Table 4:** Adult brain AVM rescreening practices for adults without symptoms of brain AVM and with initial brain imaging negative for brain AVM

Routinely Reimage (4) •Every 5 years for lifetime (1) •Every 10 years for lifetime (1) •Re-image once (2) ◦At age 18–20 years if initial screening performed at age < 18 years (1) ◦One additional negative MRI with and without contrast as an adult (1) Rescreening Modality •MRI enhanced (3) •MRI with arterial spin labeling (1) •MRA always (2), sometimes (1), never (1) obtained with MRI	Do Not Routinely Reimage (26) •Rescreen if initial MRI before year 2000 and consider rescreen if performed without contrast (1) •If MRI performed at < 18 years of age, initial MRI was a long time ago or was done without contrast, or if there is a family history of spontaneous brain bleeding from AVMs (1) •If screening prior to age 21, rescreen in 20’s (1)

Rationale for adult rescreening practices for brain AVMs are found in Table 5. Of the four who do rescreen adults for detection of new brain AVMs, two do so to reassure patients, and two cited literature or a personal experience with a patient developing a new brain AVM. The most common reason among the 26 who did not rescreen was a minimal risk of development of new brain AVMs in adults (21/26, 80.8%). Nine centers indicated that in the practitioner’s or center’s experience, the yield of rescreening in adults with previous negative screening was too low, and eight indicated that the evidence for treating asymptomatic brain AVMs in adults is not sufficient to warrant rescreening if previous imaging was negative. Five cited cost considerations, one indicated difficulty with insurance approval was a consideration, four cited concerns about causing patients and families unnecessary worry, and two cited guidelines as rationale for not rescreening.

**Table 5:** Rationale for rescreening and for not rescreening for brain AVMs in adults with HHT with initial negative brain imaging and no symptoms of brain AVM

Rationale for rescreening (4 Respondents)	Rationale for not rescreening (26 Respondents)
Reassure patients (2)	Cause patient/family unnecessary worry (4)
Literature describes patients who developed new brain AVM (1)	Minimal to no risk of new brain AVM formation in ADULTS (21)
Personal experience with patient developing new brain AVM (1)	In my own experience/in my center's experience, the yield of rescreening in ADULTS with previous negative screening for brain AVM is too low (9)
Colleague had experience with patient developing new brain AVM (0)	In my own experience/in my center's experience, the evidence for treatment of asymptomatic brain AVMs in ADULTS is not sufficient to warrant rescreening in ADULTS with previous negative screening (8)
Detect brain AVM(s) that were too small to detect on initial imaging that have grown (1)	Cost considerations (5)
Other—This is the practice recommended in the treatment guidelines (1)	Difficulty with insurance approval (1)
	Other – Guidelines (2)

Respondents were able to choose more than one rationale

Rescreening practices for new brain AVMs in adults with HHT in whom initial imaging demonstrated a brain AVM but for whom follow-up imaging for the previously diagnosed brain AVM is not needed are presented in Supplemental Table 2. Ten of 30 centers (33.3%) reimage under these circumstances. Of those that rescreen adults under these circumstances, five rescreen at intervals (one every 2 years and four every 5 years). Four defer to neurology, neurosurgery, or neurointerventional specialists for determination of rescreening intervals.

### Lung AVM rescreening practices

#### Pediatric lung AVMs

Rescreening practices for children with HHT in whom initial screening was negative for lung AVM and in whom no lung AVM symptoms exist are reported in Table 6. Twenty-five of 27 centers (92.6%) that responded to the lung AVM questions rescreen, most frequently every 5 years (18/25, 72%). Eighteen of 25 (72%) rescreen with contrast echocardiography, but seven centers rescreen with other modalities including chest X-ray with pulse oximetry, chest CT, or an exercise test. Rationale for rescreening practices for pediatric lung AVMs are found in Table 7. The most common reason cited for repeat screening was literature description of patients developing new lung AVMs (19/25, 76%). All four centers that do not rescreen cited a minimal risk for development of new lung AVMs and a minimal yield of rescreening.

**Table 6:** Pediatric lung AVM rescreening practices for children without symptoms of lung AVM and with initial screening negative for lung AVM

Routinely Rescreen (25) •Every 1 year (1) ◦With pulse oximetry and symptom assessment (1) •Every 1–2 years (1) ◦With walk test (1) •Every 3 years (1) ◦With pulse oximetry and symptom assessment (1) •Every 3–5 years depending on age (1) •Every 5 years (18) ◦Until age 18 years (4) ◦Until age 20 years (1) ◦Until transition to adult care (1) ◦Lifetime (9) ▪Spaces to every 10 years at age 18–22 years with exceptions pre and post pregnancy (1) ◦Uncertain of duration (1) ◦Unspecified duration (2) ▪Screen with physical examination and pulse oximetry until age 10–12 years and then initiate contrast echocardiography every 5 years starting at age 10–12 years (1) •Once in mid adolescence/adolescence (3) Rescreening Modality •Contrast echocardiography (“bubble echo,” agitated saline) (18) ◦Pulse oximetry for < 10–12 years and contrast echocardiogram if pulse oximetry abnormal or in children > 10–12 years (1) •Chest X-ray with pulse oximetry (3) •Chest CT with contrast (2) •Cardiopulmonary exercise test (2)	Do Not Routinely Rescreen (2) •Of two who responded no to rescreening, one is a pediatric-trained expert and one is an adult-trained expert who routinely treats children with HHT

One of 28 centers that responded about pediatric care did not respond to lung AVM questions

**Table 7:** Rationale for rescreening and for not rescreening for lung AVMs in children with HHT with initial negative lung AVM screening and no symptoms of lung AVM

Rationale for Rescreening (25 Respondents)	Rationale for Not Rescreening (2 Respondents)
Reassure parents/patients (8)	Cause patient/family unnecessary worry (0)
Literature describes patients who developed new lung AVM (19)	Minimal to no risk of new lung AVM formation in CHILDREN (0)
Personal experience with patient developing new lung AVM (13)	In my own experience/in my center's experience, the yield of rescreening in CHILDREN with previous negative screening for lung AVM is too low (0)
Colleague had experience with patient developing new lung AVM (8)	In my own experience/in my center's experience, the evidence for treatment of asymptomatic lung AVMs in CHILDREN is not sufficient to warrant rescreening in CHILDREN with previous negative screening (0)
Detect lung AVM(s) that were too small to detect on initial imaging that have grown (20)	Cost considerations (0)
Other (4) •Our prospective study to assess growth and development of lung AVMs in children with HHT (1) •Growth of small lung AVMs that could become symptomatic or clinically relevant (1) •Likelihood low if negative initial screen, but contrast echocardiogram has low risks (1) •For females to be sure we know lung AVM status prior to pregnancy given substantial risks of untreated lung AVMs (1)	Difficulty with insurance approval (0)
	Need for sedation (0)
	Other (2) •In asymptomatic children with normal oxygen saturation, the first contrast echocardiogram is done at 14–15 years of age at our center. If negative at this age and child continues to be asymptomatic and has normal oxygen saturations, we do not repeat contrast echocardiography (1) •We have returned to using saturations and six-minute walk test to screen (1)

Respondents were able to choose more than one rationale. One of 28 centers that responded about pediatric care did not respond to lung AVM questions

Rescreening practices for new lung AVMs in children with HHT in whom initial screening demonstrated a lung AVM but for whom follow-up testing specifically for the previously diagnosed lung AVM is no longer required are presented in Supplemental Table 3. Twenty-two of 26 (84.6%) centers would rescreen under these circumstances, most frequently every 5 years (13/22, 59.1%). Five other centers (22.7%) would rescreen children every 2–5 years.

#### Adult lung AVMs

Rescreening practices for adults with HHT in whom initial screening was negative for lung AVM and in whom no lung AVM symptoms exist are reported in Table 8. Twenty-six of 30 adult centers (86.7%) rescreen for lung AVMs, most frequently every 5 years (18/26, 69.2%). Five of 26 centers (19.2%) rescreen every 5–10 years, and only three of 26 (11.6%) rescreen once. All 26 who rescreen utilize contrast echocardiography. Rationale for rescreening practices for adult lung AVMs are found in Table 9. The most common reasons for rescreening were detecting growth of lung AVMs that were too small to be identified on initial screening (21/26, 80.8%), literature description of patients developing new lung AVMs (21/26, 80.8%), and personal experience with a patient developing new lung AVMs (19/26, 73.1%).

**Table 8:** Adult lung AVM rescreening practices for adults without symptoms of lung AVM and with initial screening negative for lung AVM

Routinely Rescreen (26) •Every 5 years (18) ◦Until age 50 years (2) ◦Lifetime (14) ▪Until would not be amenable to treatment (1) ◦Once over certain age with many negative screens, lengthen interval (1) ◦No long-term follow-up to direct rescreening after 10 + years (1) •Every 5–10 years (4) ◦Interval in discussion with patient (1) ◦Previously 10 years but moving a bit with influence from other North American experts, prior to any planned pregnancies, immediately following any deliveries (1) ◦If elderly person, screening repeatedly negative and *ACVRL* mutation, consider extending interval to 7–10 years, also for persons with possible HHT and sequential negative screens (1) •Every 10 years for life (1) •Once in 3–5 years (3) ◦In 3–5 years (1) ◦In 5 years (2) Rescreening Modality •Contrast echocardiography (“bubble echo,” agitated saline) (26) Comments: •We screen with bubble echocardiogram, but would only proceed with a CT for stage 2–3 positive bubble echocardiograms (1) •Echocardiogram bubble is minimally invasive and safe, so screening is easier than, for instance, an MRI for brain AVM screening (1)	Do Not Routinely Rescreen (4)

**Table 9:** Rationale for rescreening and for not rescreening for lung AVMs in adults with HHT with initial negative lung AVM screening and no symptoms of lung AVM

Rationale for rescreening (26 Respondents)	Rationale for not rescreening (4 Respondents)
Reassure patients (8)	Cause patient/family unnecessary worry (1)
Literature describes patients who developed new lung AVM (20)	Minimal to no risk of new lung AVM formation in ADULTS (4)
Personal experience with patient developing new lung AVM (19)	In my own experience/in my center's experience, the yield of rescreening in ADULTS with previous negative screening for lung AVM is too low (4)
Colleague had experience with patient developing new lung AVM (2)	In my own experience/in my center's experience, the evidence for treatment of asymptomatic lung AVMs in ADULTS is not sufficient to warrant rescreening in ADULTS with previous negative screening (2)
Detect lung AVM(s) that were too small to detect on initial imaging that have grown (21)	Cost considerations (1)
Other (6) •Guideline recommendation (2) •Expert consensus opinion/practice (1) •Bubble echocardiogram can give false negative studies, especially if poor windows or technical challenges with administration/imaging of contrast material (1) •Pregnancy concerns (pre and post) (1) •Also provsides info on evaluation for lung hypertension and high cardiac output state (1)	Difficulty with insurance approval (0)
	Other (0)

Respondents were able to choose more than one rationale

Rescreening practices for new lung AVMs in adults with HHT in whom initial screening demonstrated a lung AVM but for whom follow-up testing specifically for the previously diagnosed lung AVM is no longer required are presented in Supplemental Table 4. Twenty-six of 30 adult centers (86.7%) would rescreen under these circumstances, most commonly every 5 years (21/26, 80.8%).

## Discussion

In this survey of North American HHT CoEs, we evaluated rescreening practices for brain and lung AVMs as well as their rationale. Among CoEs that treat children with HHT, we found that nearly 90% routinely rescreen children for brain AVMs and over 90% rescreen for lung AVMs when initial screening is negative. Among CoEs that treat adults with HHT, over 85% rescreen for lung AVMs when initial screening is negative, but fewer than 15% of centers rescreen for brain AVMs. These four rescreening scenarios (pediatric and adult brain AVM, pediatric and adult lung AVM) are discussed below, but overall, differences in rescreening between children and adults appear to reflect concern about missing new or growing AVMs in children (more than adults) as well as the controversy regarding treatment of unruptured brain AVMs in adults. The variability in rescreening practices, which also extends to frequency and modality of rescreening, highlights the need for additional longitudinal studies that carefully collect information on outcomes and treatments in additional to imaging repositories for central review.

With respect to brain AVM rescreening in children, 89.3% of centers rescreen, which is approximately the same percentage of centers who reported adherence to guideline-recommended initial screening for brain AVMs in a previously published survey study [6]. No respondent reported avoiding rescreening children for brain AVMs due to concerns about causing worry for the family, cost, insurance approval, or need for sedation, the latter of which is a concern cited by the European VASCERN group and an element of the group’s rationale for not recommending initial screening for brain AVMs among children with HHT [12]. In this survey, the most commonly cited rationale for rescreening children who had initial negative screening was evaluation for growth of brain AVMs that were too small to be detected on initial imaging. Reassurance for families was also a common reason for rescreening among children. While reports of de novo brain AVM formation are scarce [7–10], one study found that three of 52 children (5.8%) with HHT developed de novo brain AVMs, one of whom presented with an intracerebral hemorrhage [7]. In the current study, among the 25 centers that rescreen children for brain AVMs, over half cited literature that described new development of brain AVMs among patients with HHT. Over one-third of respondents cited personal experience with a patient developing a new brain AVM, and many knew of a colleague who cared for a child who developed a new brain AVM, both of which may indicate that cases of de novo brain AVM formation among children with HHT are underreported in the literature (given the paucity of cases reported in the literature), leading to an underestimate of the risk for new brain vascular malformations among children with HHT. This possibility further underscores the need for more studies.

In contrast to rescreening practices for brain AVMs in children, only 13% of CoEs rescreen for brain AVMs in adults in whom initial screening was negative. This mostly reflects thinking that that evidence for treating asymptomatic brain AVMs is low and that little risk of de novo brain AVM development exists among adults. However, a publication from the Brain Vascular Malformation Consortium HHT project confirmed a case of an adult who developed a new brain AVM [8]. As noted in Table 5, only one expert who cares for adults with HHT cited personal experience with an adult patient who developed a de novo brain AVM. Considering that adult centers follow more patients with HHT than pediatric centers, the fact that 36% of pediatric respondents who rescreen had personal experience with a pediatric patient developing a new brain AVM may indicate that de novo brain AVM formation may mostly occur in the pediatric age group. However, this experience among pediatric experts may merely reflect the practice of rescreening at most pediatric programs and therefore detection of new brain AVMs, whereas the frequency of de novo brain AVMs among adults with HHT may be underestimated given that most adults are not rescreened if initial imaging was negative. Underreporting of de novo brain AVMs among adults with HHT may also occur, so it is important that cases of adults with HHT and de novo brain AVMs be collected and published, preferably in the setting of longitudinal cohorts. More information will help define risk and refine guidelines. Adult patients should be made aware that even if small, there is at least some risk of developing new brain AVMs, thereby permitting patients to have an active role in decision making with regard to rescreening preferences.

With respect to lung AVMs, over 90% of centers rescreen children, which is similar to the rescreening practices among the adult centers surveyed (87% of centers rescreen adults). The frequency of rescreening for lung AVMs, particularly among adult experts, reflects the common concern that small lung AVMs may grow larger as well as the number of experts (19 adult, 13 pediatric), who have had personal experience with a patient developing new lung AVMs. Consistent with the prior survey by Kilian et al., all centers that rescreen adults for lung AVMs use contrast echocardiography [6], but screening practices for children were more varied. Seventy-two percent of centers that evaluate children rescreen with contrast echocardiography, with the remaining utilizing at least three other methods. Notably, several centers commented that they do not perform initial screening for lung AVMs in children until they reach adolescence if the pulse oximetry readings are normal. This practice of delayed screening based on pulse oximetry demonstrates practice variability despite the 2nd International Guidelines for the Diagnosis and Management of HHT, in which the expert panel recommended screening for lung AVMs in asymptomatic children with HHT or at risk for HHT at the time of presentation or diagnosis [4]. Practice differences are not surprising and reflect some of the disagreements among experts in HHT in areas with less evidence, further supporting the need for additional longitudinal studies with imaging available for central review.

This survey study has several limitations. First, each center was permitted one response for adult care and one response for pediatric care. However, many centers have several experts who have input on care and rescreening, and individual differences may not have been captured. Additionally, the survey did not rank rationale for rescreening and not rescreening, so the single most important factor for each respondent’s decisions is not reflected. It is also possible that additional factors that limit rescreening were not identified through the survey, though there was an “other” option with space for respondents to write additional comments. Patient preferences and potential barriers that patients may face for returning to HHT CoEs were not evaluated in this study. Finally, this study reflects practices at North American HHT CoEs, which may be different from those of other practice settings. However, our goal was to report the standard practices at expert, accredited centers given the rarity of the disease and the need for expertise in the patients’ care.

## Conclusions

This study provides insight into current rescreening practices for patients who have a lifelong genetic disease that is often dynamic. The long median experience of the practitioners indicates that the respondents are knowledgeable about the patients for whom they care and are therefore an excellent barometer of current practices. However, it is clear that additional studies are required to improve rescreening rationale and to identify those patients who would benefit from rescreening as well as those who do not need additional testing.

## Supplementary Information

Additional file 1.

Additional file 2.

## Data Availability

The anonymised dataset used and analysed during the current study is available at https://curehht.org/ojrd-hht-center-of-excellence-rescreening-survey-brain-lung-avms/.

## Abbreviations

HHT: Hereditary hemorrhagic telangiectasias
AVM: Arteriovenous malformation
CoE: Center of excellence
MRI: Magnetic resonance imaging
