# Supplementary material for: Brain and lung arteriovenous malformation rescreening practices for children and adults with hereditary hemorrhagic telangiectasia
Source: Orphanet J Rare Dis. 2024 Nov 9;19:421. doi: 10.1186/s13023-024-03402-8 (PMC11549847; doi:10.1186/s13023-024-03402-8)
Supplement: Supplementary file 1 — Additional file 1. [file 13023_2024_3402_MOESM1_ESM.pdf]

Survey: Re-Screening Practices for Brain AVMs and Pulmonary AVMs in Patients with HHT

Dear Center Directors and Co-Directors,

We are hoping to learn about re-screening practices for brain AVMs and pulmonary AVMs in CHILDREN (age <18 years) and ADULTS (age >=18 years) with HHT. We very much appreciate your time in filling out this survey about your practices, which will take about 15 minutes.

Please contact Lauren Beslow at beslow@chop.edu with any questions.

Thank you for your time and consideration.

Best regards,

Dr. Lauren Beslow and Dr. Marie Faughnan

Survey: Re-Screening Practices for Brain AVMs and Pulmonary AVMs in Patients with HHT

Respondent Questions

* 1. Which HHT Clinical Center do you work at?

* 2. What is your name?

* 3. Are you the Center Director or Co-Director/Associate Director?

- Yes
- No

* 4. How many years have you (the responder) been evaluating/ managing HHT patients?

HHT Care for Children at Your Center

* 5. Do you manage CHILDREN (age<18 years) with HHT?

(If you manage some CHILDREN, please say yes. Please also provide name/email later in survey of provider who manages most CHILDREN at your center.)

- ◯ Yes
- ◯ No

HHT Care for Children at Your Center

6. Approximately how many CHILDREN (< 18 years) with HHT does your center follow?

7. If you manage CHILDREN (<18 years) with HHT but DO NOT manage ADULTS (>=18 years) with HHT, what is the **name** of the person who manages ADULTS (>=18 years) with HHT at your center? (We will send a survey to your colleague who manages ADULTS!)

8. If you manage CHILDREN (<18 years) with HHT but DO NOT manage ADULTS (>=18 years) with HHT, what is the **email** of the person who manages ADULTS (>=18 years) with HHT at your center? (We will send a survey to your colleague who manages ADULTS!)

Survey: Re-Screening Practices for Brain AVMs and Pulmonary AVMs in Patients with HHT

RE-SCREENING PRACTICES: ASYMPTOMATIC CHILDREN with Initially NEGATIVE Brain AVM Screening

The next questions are about your current practice regarding repeating brain imaging (re-screening) for CHILDREN (age<18 years) with HHT who had NEGATIVE initial brain imaging (i.e., no brain AVM on initial MRI screening)

* 9. For CHILDREN (<18 years) with HHT, do you routinely recommend re-imaging (re-screening) for brain AVMs in children with NO symptoms of brain AVM if initial brain imaging was NEGATIVE for brain AVM?

- ◯ Yes
- ◯ No

Survey: Re-Screening Practices for Brain AVMs and Pulmonary AVMs in Patients with HHT

RE-SCREENING PRACTICES: ASYMPTOMATIC CHILDREN with Initially NEGATIVE Brain AVM Screening

10. If you answered YES that you do re-image (re-screen) CHILDREN (<18 years) with HHT for brain AVMs if initial imaging for brain AVMs was NEGATIVE and they are asymptomatic of brain AVM, at what interval do you repeat imaging (re-screen) for brain AVM?

- ○ Re-image once if initial screening performed at age <1 year
- ○ Re-image once if initial screening performed at age <2 years
- ○ Re-image once if initial screening performed at age <5 years
- ○ Re-image once no matter the age at initial screening
- ○ Every 2 years
- ○ Every 5 years
- ○ Every 7 years
- ○ Every 10 years
- ○ Other (please specify)

Survey: Re-Screening Practices for Brain AVMs and Pulmonary AVMs in Patients with HHT

RE-SCREENING PRACTICES: ASYMPTOMATIC CHILDREN with Initially NEGATIVE Brain AVM Screening

11. If you re-image (re-screen) ONCE, at what age, age range, or time from initial image do you re-image (re-screen)?

RE-SCREENING PRACTICES: ASYMPTOMATIC CHILDREN with Initially NEGATIVE Brain AVM Screening

12. If you re-image (re-screen) at intervals, until what age do you repeat imaging to re-screen for brain AVM?

- Until age 15 years
- Until age 18 years
- Until age 20 years
- Until age 25 years
- Lifetime
- Other (please specify)

RE-SCREENING PRACTICES: ASYMPTOMATIC CHILDREN with Initially NEGATIVE Brain AVM Screening

13. Which re-imaging (re-screening) test do you order/recommend MOST FREQUENTLY in CHILDREN to re-screen for brain AVMs?

- ◯ MRI (un-enhanced or non-contrast)
- ◯ MRI (enhanced or with contrast)
- ◯ CT (enhanced or with contrast)
- ◯ Conventional angiogram
- ◯ Other (please specify)

14. If you re-image (re-screen) CHILDREN for brain AVMs with MRI (either enhanced or un-enhanced), do you also obtain MRA [MR angiogram]?

- ◯ Always
- ◯ Sometimes
- ◯ Never
- ◯ Unsure

Additional Comments

15. If you DO re-image (re-screen) asymptomatic CHILDREN for brain AVMs if initial screening was negative for brain AVM, why do you re-image (re-screen)? (Check all that apply)

- [ ] Reassure patients/parents
- [ ] Literature describes patients who developed new brain AVM
- [ ] Personal experience with patient developing new brain AVM
- [ ] Colleague had experience with patient developing new brain AVM
- [ ] Detect brain AVM(s) that were too small to detect on initial imaging that have grown
- [ ] Other (please specify)

RE-SCREENING PRACTICES: ASYMPTOMATIC CHILDREN with Initially NEGATIVE Brain AVM Screening

16. If you DO NOT re-image (re-screen) CHILDREN for brain AVMs if initial screening was negative for brain AVM, why do you not re-image (re-screen)? (Check all that apply)

- [ ] Do not want to cause patient/family unnecessary worry
- [ ] Minimal to no risk of new brain AVM formation in CHILDREN
- [ ] In my own experience/in my center's experience, the yield of re-screening in CHILDREN with previous negative screening for brain AVM is too low
- [ ] In my own experience/in my center's experience, the evidence for treatment of asymptomatic brain AVMs in CHILDREN is not sufficient to warrant re-screening in CHILDREN with previous negative screening
- [ ] Cost considerations
- [ ] Difficulty with insurance approval
- [ ] Need for sedation
- [ ] Other (please specify)

Survey: Re-Screening Practices for Brain AVMs and Pulmonary AVMs in Patients with HHT

RE-SCREENING PRACTICES: CHILDREN WITH KNOWN BRAIN AVM

These questions are about children WITH brain AVM(s)

17. For CHILDREN with a history of brain AVM(s), would you re-image (re-screen) for new (de novo) brain AVM(s), even if you are not planning follow-up imaging for the previously diagnosed brain AVM(s)?

- ○ Yes
- ○ No

RE-SCREENING PRACTICES: CHILDREN WITH KNOWN BRAIN AVM

18. If you answered YES that you do re-image (re-screen) CHILDREN with a history of brain AVM(S) for new (de novo) brain AVM(s), at what interval do you repeat imaging (re-screen)?

- ○ Re-image once if initial imaging performed at age < 1 year
- ○ Re-image once if initial screening performed at age < 2 years
- ○ Re-image once if initial screening performed at age < 5 years
- ○ Every 2 years
- ○ Every 5 years
- ○ Every 7 years
- ○ Every 10 years
- ○ Other (please specify)

RE-SCREENING PRACTICES: CHILDREN WITH KNOWN BRAIN AVM

19. If you re-image (re-screen) CHILDREN with a history of brain AVM(s) for new (de novo) brain AVM(s) ONCE, at what age, age range, or time from initial image do you re-image (re-screen)?

Survey: Re-Screening Practices for Brain AVMs and Pulmonary AVMs in Patients with HHT

RE-IMAGING PRACTICES: CHILDREN WITH KNOWN BRAIN AVM

20. If you re-image (re-screen) CHILDREN with a history of brain AVM(s) at intervals, until what age do you repeat imaging to re-screen for new (de novo) brain AVM?

- ◯ Until age 15 years
- ◯ Until age 18 years
- ◯ Until age 20 years
- ◯ Until age 25 years
- ◯ Lifetime
- ◯ Other (please specify)

Survey: Re-Screening Practices for Brain AVMs and Pulmonary AVMs in Patients with HHT

Any Additional Comments: BRAIN AVMs in CHILDREN

21. Additional Comments (Write In)

Survey: Re-Screening Practices for Brain AVMs and Pulmonary AVMs in Patients with HHT

RE-SCREENING PRACTICES: ASYMPTOMATIC CHILDREN with Initially NEGATIVE Pulmonary AVM Screening

The next questions are about your current practice regarding repeating studies to evaluate for pulmonary AVMs (RE-screening) for CHILDREN (age<18 years) with HHT who had NEGATIVE initial pulmonary AVM screening (i.e., no pulmonary AVM on initial screening)

* 22. For CHILDREN (<18 years) with HHT, do you routinely recommend re-screening for pulmonary AVMs in children with NO symptoms of pulmonary AVM if initial screening was NEGATIVE for pulmonary AVM?

- ◯ Yes
- ◯ No

Survey: Re-Screening Practices for Brain AVMs and Pulmonary AVMs in Patients with HHT

RE-SCREENING PRACTICES: ASYMPTOMATIC CHILDREN with Initially NEGATIVE Pulmonary AVM Screening

23. If you answered YES that you do re-screen CHILDREN (< 18 years) with HHT for pulmonary AVMs if initial screening for pulmonary AVMs was negative and they are asymptomatic of pulmonary AVM, at what interval do you re-screen for pulmonary AVM?

- Re-screen once if initial screening performed at age <1 year
- Re-screen once if initial screening performed at age <2 years
- Re-screen once if initial screening performed at age <5 years
- Re-screen once no matter the age at initial screening
- Every 2 years
- Every 5 years
- Every 7 years
- Every 10 years
- Other (please specify)

Survey: Re-Screening Practices for Brain AVMs and Pulmonary AVMs in Patients with HHT

RE-SCREENING PRACTICES: ASYMPTOMATIC CHILDREN with Initially NEGATIVE Pulmonary AVM Screening

24. If you re-screen ONCE, at what age, age range, or time from initial screen do you re-screen?

Survey: Re-Screening Practices for Brain AVMs and Pulmonary AVMs in Patients with HHT

RE-SCREENING PRACTICES: ASYMPTOMATIC CHILDREN with Initially NEGATIVE Pulmonary AVM Screening

25. If you rescreen at intervals, until what age do you re-screen for pulmonary AVM?

- Until age 15 years
- Until age 18 years
- Until age 20 years
- Until age 25 years
- Lifetime
- Other (please specify)

Survey: Re-Screening Practices for Brain AVMs and Pulmonary AVMs in Patients with HHT

RE-SCREENING PRACTICES: ASYMPTOMATIC CHILDREN with Initially NEGATIVE Pulmonary AVM Screening

26. Which re-screening test do you order/recommend MOST FREQUENTLY in CHILDREN to re-screen for pulmonary AVMs?

- ◯ Contrast echocardiography (“bubble” echo, agitated saline injection)
- ◯ CT chest (un-enhanced or non-contrast)
- ◯ CT chest (enhanced or with contrast)
- ◯ Chest x-ray and pulse oximetry
- ◯ Cardiopulmonary exercise test

27. If you DO re-screen CHILDREN for pulmonary AVMs if initial screening was negative for pulmonary AVM, why do you re-screen? (Check all that apply)

- ☐ Reassure patients
- ☐ Literature describes patients who developed new pulmonary AVM
- ☐ Personal experience with patient developing new pulmonary AVM
- ☐ Colleague had experience with patient developing new pulmonary AVM
- ☐ Detect pulmonary AVM(s) that were too small to detect on initial imaging that have grown
- ☐ Other (please specify)

Survey: Re-Screening Practices for Brain AVMs and Pulmonary AVMs in Patients with HHT

RE-SCREENING PRACTICES: ASYMPTOMATIC CHILDREN with Initially NEGATIVE Pulmonary AVM Screening

28. If you DO NOT re-screen CHILDREN for pulmonary AVMs if initial screening was negative for pulmonary AVM, why do you not re-screen? (Check all that apply)

- [ ] Cause patient/family unnecessary worry
- [ ] Minimal to no risk of new pulmonary AVM formation in CHILDREN
- [ ] In my own experience/in my center's experience, the yield of re-screening in CHILDREN with previous negative screening for pulmonary AVM is too low
- [ ] In my own experience/in my center's experience, the evidence for treatment of asymptomatic pulmonary AVMs in CHILDREN is not sufficient to warrant re-screening in CHILDREN with previous negative screening
- [ ] Cost considerations
- [ ] Difficulty with insurance approval
- [ ] Need for sedation
- [ ] Other (please specify)

RE-SCREENING PRACTICES: CHILDREN WITH KNOWN PULMONARY AVM

These questions are about children WITH pulmonary AVM(s)

29. For CHILDREN with a history of pulmonary AVM(s), would you re-screen for new (de novo) pulmonary AVM(s), even if you are not planning follow-up testing for the previously diagnosed pulmonary AVM(s)?

- ◯ Yes
- ◯ No

RE-SCREENING PRACTICES: CHILDREN WITH KNOWN PULMONARY AVM

30. If you answered YES that you do re-screen CHILDREN with a history of pulmonary AVM(s) for new (de novo) pulmonary AVM(s), at what interval to you repeat screening?

- ◯ Re-screen once if initial screening performed at age < 1 year
- ◯ Re-screen once if initial screening performed at age < 2 years
- ◯ Re-screen once if initial screening performed at age < 5 years
- ◯ Every 2 years
- ◯ Every 5 years
- ◯ Every 7 years
- ◯ Every 10 years
- ◯ Other (please specify)

RE-SCREENING PRACTICES: CHILDREN WITH KNOWN PULMONARY AVM

31. If you re-screen CHILDREN with a history of pulmonary AVM(s) for new (de novo) AVM(s) ONCE, at what age, age range, or time from initial image do you re-screen?

RE-SCREENING PRACTICES: CHILDREN WITH KNOWN PULMONARY AVM

32. If you re-screen CHILDREN with a history of pulmonary AVM(s) at intervals, until what age do you re-screen for new (de novo) pulmonary AVM?

- Until age 15 years
- Until age 18 years
- Until age 20 years
- Until age 25 years
- Lifetime
- Other (please specify)

Survey: Re-Screening Practices for Brain AVMs and Pulmonary AVMs in Patients with HHT

Any Additional Comments: PULMONARY AVMs in CHILDREN

33. Additional Comments (Write In)

HHT Care for Adults at Your Center

* 34. Do you manage ADULTS (age>=18 years) with HHT?

(If you manage some ADULTS, please say yes. Please also provide name/email earlier in survey of provider who manages most ADULTS at your center.)

- ○ Yes
- ○ No

HHT Care for Adults at Your Center

35. Approximately how many ADULT (>=18 years) HHT patients total does your center follow?

36. If you manage ADULTS (>=18 years) with HHT but DO NOT manage CHILDREN (<18 years) with HHT, what is the **name** of the person who manages CHILDREN (<18 years) with HHT at your center? (We will send a survey to your colleague who manages CHILDREN!)

37. If you manage ADULTS (>=18 years) with HHT but DO NOT manage CHILDREN (<18 years) with HHT, what is the **email** of the person who manages CHILDREN (<18 years) with HHT at your center? (We will send a survey to your colleague who manages CHILDREN!)

Survey: Re-Screening Practices for Brain AVMs and Pulmonary AVMs in Patients with HHT

RE-SCREENING PRACTICES: ASYMPTOMATIC ADULTS with Initially NEGATIVE Brain AVM Screening

The next questions are about your current practice regarding repeating brain imaging (Re-screening) for ADULTS (age>=18 years) with HHT who had NEGATIVE initial brain screening (i.e., no brain AVM on initial screening)

* 38. For ADULTS (>=18 years) with HHT, do you routinely recommend re-imaging (re-screening) for brain AVMs in adults with NO symptoms of brain AVM if initial brain imaging was NEGATIVE for brain AVM?

- ◯ Yes
- ◯ No

RE-SCREENING PRACTICES: ASYMPTOMATIC ADULTS with Initially NEGATIVE Brain AVM Screening

39. If you answered YES that you do re-image (re-screen) ADULTS (>=18 years) for brain AVMs if initial imaging for brain AVMs was negative and they are asymptomatic of brain AVM, at what interval do you repeat imaging (re-screen) for brain AVM?

- ◯ Re-image once only if initial screening performed at age <18 years
- ◯ Re-image once no matter age at initial screening
- ◯ Every 2 years
- ◯ Every 5 years
- ◯ Every 7 years
- ◯ Every 10 years
- ◯ Other (please specify)

Survey: Re-Screening Practices for Brain AVMs and Pulmonary AVMs in Patients with HHT

RE-SCREENING PRACTICES: ASYMPTOMATIC ADULTS with Initially NEGATIVE Brain AVM Screening

40. If you re-image (re-screen) ONCE, at what age, age range, or time from initial image do you re-image (re-screen)?

Survey: Re-Screening Practices for Brain AVMs and Pulmonary AVMs in Patients with HHT

RE-SCREENING PRACTICES: ASYMPTOMATIC ADULTS with Initially NEGATIVE Brain AVM Screening

41. If you re-image (re-screen) at intervals, until what age do you repeat imaging to re-screen for brain AVM?

- ○ Until age 20 years
- ○ Until age 25 years
- ○ Until age 30 years
- ○ Until age 40 years
- ○ Until age 50 years
- ○ Lifetime
- ○ Other (please specify)

Survey: Re-Screening Practices for Brain AVMs and Pulmonary AVMs in Patients with HHT

RE-SCREENING PRACTICES: ASYMPTOMATIC ADULTS with Initially NEGATIVE Brain AVM Screening

42. Which re-imaging (re-screening) test do you order/recommend MOST FREQUENLY in ADULTS to re-screen for brain AVMs?

- ◯ MRI (un-enhanced or non-contrast)
- ◯ MRI (enhanced or with contrast)
- ◯ CT (enhanced or with contrast)
- ◯ Conventional angiogram
- ◯ Other (please specify)

43. If you re-image (re-screen) ADULTS for brain AVMs with MRI (either enhanced or un-enhanced), do you also obtain MRA [MR angiogram]?

- ◯ Always
- ◯ Sometimes
- ◯ Never
- ◯ Unsure

Comment (write in)

44. If you DO re-image (re-screen) ADULTS for brain AVMs if initial screening was negative for brain AVM, why do you re-image (re-screen)? (Check all that apply)

- [ ] Reassure patients
- [ ] Literature describes patients who developed new brain AVM
- [ ] Personal experience with patient developing new brain AVM
- [ ] Colleague had experience with patient developing new brain AVM
- [ ] Detect brain AVM(s) that were too small to detect on initial imaging that have grown
- [ ] Other (please specify)

Survey: Re-Screening Practices for Brain AVMs and Pulmonary AVMs in Patients with HHT

RE-SCREENING PRACTICES: ASYMPTOMATIC ADULTS with Initially NEGATIVE Brain AVM Screening

45. If you DO NOT re-image (re-screen) ADULTS for brain AVMs if initial screening was negative for brain AVM, why do you not re-image (re-screen)? (Check all that apply)

- [ ] Cause patient/family unnecessary worry
- [ ] Minimal to no risk of new brain AVM formation in ADULTS
- [ ] In my own experience/in my center's experience, the yield of re-screening in ADULTS with previous negative screening for brain AVM is too low
- [ ] In my own experience/in my center's experience, the evidence for treatment of asymptomatic brain AVMs in ADULTS is not sufficient to warrant re-screening in ADULTS with previous negative screening
- [ ] Cost considerations
- [ ] Difficulty with insurance approval
- [ ] Other (please specify)

RE-SCREENING PRACTICES: ADULTS WITH KNOWN BRAIN AVM

These questions are about adults with brain AVM(s)

46. For ADULTS with a history of brain AVM(s), would you re-image (re-screen) for new (de novo) brain AVM(s), even if you are not planning follow-up imaging for the previously diagnosed brain AVM(s)?

- ◯ Yes
- ◯ No

RE-SCREENING PRACTICES: ADULTS WITH KNOWN BRAIN AVM

47. If you answered YES that you do re-image (re-screen) ADULTS with a history of brain AVM(S) for new (de novo) brain AVM(s), at what interval do you repeat imaging (re-screen)?

- ◯ Re-image once if initial imaging performed at age <18 years
- ◯ Re-image once no matter age at initial screening
- ◯ Every 2 years
- ◯ Every 5 years
- ◯ Every 7 years
- ◯ Every 10 years
- ◯ Other (please specify)

RE-SCREENING PRACTICES: ADULTS WITH KNOWN BRAIN AVM

48. If you do re-image (re-screen) ADULTS with a history of brain AVM(s) ONCE, at what age, age range, or time from initial image do you re-image (re-screen)?

Survey: Re-Screening Practices for Brain AVMs and Pulmonary AVMs in Patients with HHT

RE-SCREENING PRACTICES: ADULTS WITH KNOWN BRAIN AVM

49. If you re-image (re-screen) ADULTS with a history of brain AVM(s) at intervals, until what age do you repeat imaging to re-screen for brain AVM?

- Until age 20 years
- Until age 25 years
- Until age 30 years
- Until age 40 years
- Until age 50 years
- Lifetime
- Other (please specify)

Any Additional Comments: BRAIN AVMs in ADULTS

50. Additional Comments (Write In)

RE-SCREENING PRACTICES: ASYMPTOMATIC ADULTS with Initially NEGATIVE Pulmonary AVM Screening

The next questions are about your current practice regarding repeating studies to evaluate for pulmonary AVMs (RE-screening) for ADULTS (age>=18 years) with HHT who had NEGATIVE initial pulmonary AVM screening (i.e., no pulmonary AVM on initial screening)

* 51. For ADULTS (>= 18 years) with HHT, do you routinely recommend re-screening for pulmonary AVMs in adults with NO symptoms of pulmonary AVM if initial screening was NEGATIVE for pulmonary AVM?

◯ Yes

◯ No

Survey: Re-Screening Practices for Brain AVMs and Pulmonary AVMs in Patients with HHT

RE-SCREENING PRACTICES: ASYMPTOMATIC ADULTS with Initially NEGATIVE Pulmonary AVM Screening

52. If you answered YES that you do re-screen ADULTS (>= 18 years) with HHT for pulmonary AVMs if initial screening for pulmonary AVMs was negative and they are asymptomatic of pulmonary AVM, at what interval do you re-screen for pulmonary AVM?

- Re-screen once if initial screening performed at age <18 years
- Re-screen once no matter age at initial screening
- Every 2 years
- Every 5 years
- Every 7 years
- Every 10 years
- Other (please specify)

RE-SCREENING PRACTICES: ASYMPTOMATIC ADULTS with Initially NEGATIVE Pulmonary AVM Screening

53. If you re-screen once, is there a particular age, age range, or time from initial screen at which you re-screen?

RE-SCREENING PRACTICES: ASYMPTOMATIC ADULTS with Initially NEGATIVE Pulmonary AVM Screening

54. If you rescreen at intervals, until what age do you re-screen for pulmonary AVM?

- Until age 20 years
- Until age 25 years
- Until age 30 years
- Until age 40 years
- Until age 50 years
- Lifetime
- Other (please specify)

RE-SCREENING PRACTICES: ASYMPTOMATIC ADULTS with Initially NEGATIVE Pulmonary AVM Screening

55. Which re-screening test do you order/recommend MOST FREQUENTLY in ADULTS to re-screen for pulmonary AVMs?

- ○ Contrast echocardiography (“bubble” echo, agitated saline injection)
- ○ CT chest (un-enhanced or non-contrast)
- ○ CT chest (enhanced or with contrast)
- ○ Chest x-ray and pulse oximetry
- ○ Cardiopulmonary exercise test

56. If you DO re-screen ADULTS for pulmonary AVMs if initial screening was negative for pulmonary AVM, why do you re-screen? (Check all that apply)

- ☐ Reassure patients
- ☐ Literature describes patients who developed new pulmonary AVM
- ☐ Personal experience with patient developing new pulmonary AVM
- ☐ Colleague had experience with patient developing new pulmonary AVM
- ☐ Detect pulmonary AVM(s) that were too small to detect on initial imaging that have grown
- ☐ Other (please specify)

RE-SCREENING PRACTICES: ASYMPTOMATIC ADULTS with Initially NEGATIVE Pulmonary AVM Screening

57. If you DO NOT re-screen ADULTS for pulmonary AVMs if initial screening was negative for pulmonary AVM, why do you not re-screen? (Check all that apply)

- ☐ Cause patient/family unnecessary worry
- ☐ Minimal to no risk of new pulmonary AVM formation in ADULTS
- ☐ In my own experience/in my center's experience, the yield of re-screening in ADULTS with previous negative screening for pulmonary AVM is too low
- ☐ In my own experience/in my center's experience, the evidence for treatment of asymptomatic pulmonary AVMs in ADULTS is not sufficient to warrant re-screening in ADULTS with previous negative screening
- ☐ Cost considerations
- ☐ Difficulty with insurance approval
- ☐ Other (please specify)

Survey: Re-Screening Practices for Brain AVMs and Pulmonary AVMs in Patients with HHT

RE-SCREENING PRACTICES: ADULTS WITH KNOWN PULMONARY AVM

These questions are about adults WITH pulmonary AVM(s)

58. For ADULTS with a history of pulmonary AVM(s), would you re-screen for new (de novo) pulmonary AVM(s), even if you are not planning follow-up imaging for the previously diagnosed pulmonary AVM(s)?

- ◯ Yes
- ◯ No

RE-SCREENING PRACTICES: ADULTS WITH KNOWN PULMONARY AVM

59. If you answered YES that you do re-screen ADULTS with a history of pulmonary AVM(s) for new (de novo) pulmonary AVM(s), at what interval to you re-screen?

- ◯ Re-screen once if initial screening performed at age <18 years
- ◯ Re-screen once no matter age at initial screening
- ◯ Every 2 years
- ◯ Every 5 years
- ◯ Every 7 years
- ◯ Every 10 years
- ◯ Other (please specify)

Survey: Re-Screening Practices for Brain AVMs and Pulmonary AVMs in Patients with HHT

RE-SCREENING PRACTICES: ADULTS WITH KNOWN PULMONARY AVM

60. If you re-screen ADULTS with a history of pulmonary AVM(s) ONCE, at what age, age range, or time from initial screen do you re-screen?

Survey: Re-Screening Practices for Brain AVMs and Pulmonary AVMs in Patients with HHT

RE-SCREENING PRACTICES: ADULTS WITH KNOWN PULMONARY AVM

61. If you re-screen ADULTS with a history of pulmonary AVM(s) at intervals, until what age do you re-screen for pulmonary AVM?

- Until age 20 years
- Until age 25 years
- Until age 30 years
- Until age 40 years
- Until age 50 years
- Lifetime
- Other (please specify)

Survey: Re-Screening Practices for Brain AVMs and Pulmonary AVMs in Patients with HHT

Any Additional Comments: PULMONARY AVMs in ADULTS

62. Additional Comments (Write In)
