# Supplementary material for: Brain and lung arteriovenous malformation rescreening practices for children and adults with hereditary hemorrhagic telangiectasia
Source: Orphanet J Rare Dis. 2024 Nov 9;19:421. doi: 10.1186/s13023-024-03402-8 (PMC11549847; doi:10.1186/s13023-024-03402-8)
Supplement: Supplementary file 2 — Additional file 2. [file 13023_2024_3402_MOESM2_ESM.docx]

**Supplemental Rescreening Practices Tables**

**Supplemental Table 1.** Pediatric Brain AVM Rescreening Practices to Evaluate for *De Novo* Brain AVMs for Children with HHT and History of Brain AVM that No Longer Require Follow-up Imaging

| **Routinely Reimage (23)**   - Every 2 years (1) - Every 5 years (6) - Until age 18 years (4) - Until age 25 years (1) - Lifetime (1) - Every 10 years (3) - Until age 18 years, (1) - At age 6 months, age 10 years, age 18-20 years, perhaps more frequent with neurosurgery input (1) - No duration specified (1) - Once or twice (8) - In 2-3 years - difficult to assess asymptomatic in child with known brain AVM (1) - If initial imaging performed at age <5 years (2) - If initial imaging performed prior to puberty (1) - In mid adolescence (1) - Puberty and age 17-18 years (1) - At age 18 years (1) - No qualifier specified (1) - Defer to neurology, neurosurgery, and/or neurointerventional radiology (4) - Varies between years versus less often based on concern for size, complexity, symptoms (1) - Would defer to my regular guidelines, but my neurosurgery colleagues always reimage (1) - Other (1) - Varies with initial imaging (1)   **Comments:** Screening becomes more individualized in children with treatment of prior brain AVM and screening intervals might change in children with indeterminate vascular lesions, such as an enhancing lesion that is too small to characterize | **Do Not Routinely Reimage (3)**   - Children with known brain AVMs are followed by our specialist team of interventional radiologists and neurosurgeons (1) - Of three who responded no to rescreening, two are pediatric-trained experts and one is an adult-trained expert who routinely treats children with HHT |
| --- | --- |

Two of 28 centers that responded about pediatric care did not respond to these questions.

**Supplemental Table 2.** Adult Brain AVM Rescreening Practices to Evaluate for *De Novo* Brain AVMs for Adults with HHT and History of Brain AVM that No Longer Require Follow-up Imaging

| **Routinely Reimage (10)**   - Every 2 years for life (1) - Every 5 years (4) - Lifetime (3) - Until age 75 years (1) - Once if initial imaging performed at age <18 years (1) - Other (4) - Defer to neurology, neurosurgery, and/or neurointerventional radiology (4) - At least once if imaging at <18 years (1) - 1 year after surgery and in longer intervals thereafter, every 1-3 years (1) | **Do Not Routinely Reimage (20)**  **Comments**   - Very important topic; we have several HHT patients with lung arterial hypertension on lung vasodilators and it would be interesting to know whether these drugs change the appearance or growth of brain AVMs (1) - My understanding is that new brain AVMs do not develop in adulthood in HHT patients and therefore continued brain AVM screening is not necessary even in patients that have a history of brain AVM(s). The thinking is that the existing brain AVM(s) developed in utero or in early life. Happy to be corrected/learn/change practice if my understanding is incorrect (1) - Depends on a number of factors (1) - Re-imaging is sometimes driven by patient preference (i.e., some patients ask to be rescreened) (1) - Cannot think of anytime we have NOT done follow-up imaging of know brain AVM whether treated or not, so this is not a valid question (1) |
| --- | --- |

**Supplemental Table 3.** Pediatric Lung AVM Rescreening Practices to Evaluate for *De Novo* Lung AVMs for Children with HHT and History of Lung AVM that No Longer Requires Follow-up

| **Routinely Rescreen (22)**   - Every 2 years (1) - Until age 18 years (1) I try and use TTCE for rescreening, keeping in mind that it is positive at baseline. If bubbles are felt to have increased significantly, I have a lower threshold for obtaining a CT - Every 3 years (3) - Every 3-5 years (1) - If known lung AVM treated every three years, if untreated every 5 years (1) - Every 5 years (13) - Screening 1-year post-treatment to assess for reperfusion then every 5 years (1) - Until age 18 years or throughout pediatric care (6) - Lifetime (6) - With input from interventional radiology (1) - Sometimes every 2-3 years (1) - Combination of clinical assessment (saturation, six-minute walk test, symptoms) (1) - Depends on many clinical factors including patient/parent preference, patient availability, post-treatment course of known lung AVM (1) - In reality I would also be interested in growth of known AVMs (1) - Always follow up known AVMs (1) | **Do Not Routinely Rescreen (4)**   - Of four who responded no to rescreening, two are pediatric-trained experts and two are adult-trained experts who routinely treat children with HHT |
| --- | --- |

Two of 28 centers that responded about pediatric care did not respond to these lung AVM questions. TTCE, transthoracic contrast echocardiography

**Supplemental Table 4.** Adult Lung AVM Rescreening Practices to Evaluate for De Novo Lung AVMs for Adults with HHT and History of Lung AVM that No Longer Requires Follow-up

| **Routinely Rescreen (26)**   - Every 2 years for life or until no longer amenable to treatment (1) - Since follow for known lung AVM, screen for new one at same time (1) - Every 3-5 years (1) - Not screening for *de novo* lung AVM as this never happens, following existing ones and even their current recommendations are far too aggressive (1) - Every 3 years (1) - Every 5 years (21) - 1 year after embolization to screen for reperfusion then every 5 years (1) - Lifetime (13) - Varies according to size/behavior of known lung AVMs and general health status, but typically well past 50 years (1) - Denes on a number of factors (1) - Individualized, if would go after a macro-AVM then would screen (1) - Until age 50 years (1) - Until about 70 years (1) - Until about 70-80 years (1) - Unspecified duration (1)   **Comment:** rescreening after embolization treatment is likely to be done with contrast CT, with transthoracic contrast echocardiogram as adjunct (1) | **Do Not Routinely Rescreen (4)** |
| --- | --- |
